# Prognostic Value of Body Mass Index Stratified by Alcohol Drinking Status in Patients With Esophageal Squamous Cell Carcinoma

**DOI:** 10.3389/fonc.2022.769824

**Published:** 2022-02-17

**Authors:** Shao-bin Chen, Di-tian Liu, Yu-ping Chen

**Affiliations:** Department of Thoracic Surgery, Cancer Hospital of Shantou University Medical College, Shantou, China

**Keywords:** alcohol drinking, body mass index, esophageal neoplasm, prognosis, squamous cell carcinoma

## Abstract

**Background:**

The goal of this study was to investigate the prognostic value of body mass index (BMI) in patients with esophageal squamous cell carcinoma (ESCC) when stratified by alcohol drinking status.

**Methods:**

A total of 620 patients with ESCC who underwent esophagectomy were analyzed. A receiver operating characteristic curve was constructed to set the appropriate cutoff point for BMI. Alcohol drinking was divided into ever and never. Kaplan-Meier and multivariate Cox regression analyses were conducted to investigate the association between clinicopathological factors and survival.

**Results:**

The cutoff point was 18.75 kg/m^2^ for BMI. Two hundred and twenty-nine patients were ever drinkers, while the other 391 patients were never drinkers. The ever drinker group was found to have more males, longer tumor lengths, advanced pT category disease, advanced pN category disease, and lower tumor locations. However, no significant difference in BMI was found between ever drinkers and never drinkers. For ever drinkers, low BMI was significantly correlated with worse overall survival (hazard ratio = 1.690; P=0.035) and cancer-specific survival (hazard ratio = 1.763; P=0.024) than high BMI after adjusting for other factors. However, BMI was not a prognostic factor in univariate and multivariate analyses for never drinkers.

**Conclusions:**

BMI is a prognostic factor only in ever drinkers with ESCC but not in never drinkers. Further studies are needed to elucidate the mechanism underlying the effect of the interaction between BMI and alcohol consumption on the prognosis of patients with ESCC.

## Introduction

Esophageal carcinoma is one of the most common digestive system malignancies, with esophageal squamous cell carcinoma (ESCC) and esophageal adenocarcinoma (EAC) as the two main histological subtypes (1). Esophagectomy remains the most important tool for the treatment of resectable cases; however, the prognosis of patients with esophageal carcinoma is still poor.

Malnutrition is a frequent problem in cancer patients, and preoperative nutritional status has been found to be correlated with outcomes in patients with malignancies (2). Body mass index (BMI) is one of the preoperative nutritional parameters and has been found to be correlated with the survival of patients with various carcinomas (3–6). It has been reported that a lower BMI increases the risk of developing ESCC (7). However, the prognostic value of BMI in patients with ESCC after esophagectomy is still controversial (8). Some previous studies found that a lower BMI was associated with poorer survival in ESCC patients after esophagectomy (9–11); however, others found that a lower BMI had no impact on the prognosis of ESCC patients (12, 13) or even had a favorable impact (14, 15).

Alcohol abuse is a leading risk factor for death globally and has been found to be correlated with multiple carcinomas (16, 17). Globally, an estimated 741300 of all new cases of cancer in 2020 were attributable to alcohol consumption, while esophageal carcinoma was the most common alcohol-attributable cancer (18). Alcohol consumption has also been found to affect BMI in the general population (19–21). Although alcohol consumption is a well-established risk factor for multiple cancers, little is known about its influence on the prognosis of these cancers, and even less is known about the effect of the interaction between BMI and alcohol consumption on the prognosis of cancer patients. Some studies reported an adverse impact of alcohol consumption on the survival of cancer patients (22–25), whereas others could not obtain consistent results (26, 27).

Given that alcohol abuse affects BMI in the general population and that both BMI and alcohol abuse may affect the prognosis of ESCC patients, we hypothesize that the prognostic value of BMI in ESCC patients may be impacted by alcohol consumption status. However, to the best of our knowledge, no studies have investigated the effect of the interaction between BMI and alcohol consumption on the prognosis of ESCC patients after esophagectomy. Thus, in the current study, we aimed to investigate the value of BMI in patients with ESCC who underwent surgical resection when stratified by alcohol drinking status.

## Patients and Methods

### Patients

A total of 817 patients with esophageal cancer underwent esophagectomy at Shantou University Medical College Cancer Hospital between September 2014 and December 2017. Only patients with ESCC who chose surgery as their initial treatment were enrolled in this study. This study was approved by an independent ethics committee at our hospital. All patients provided informed consent.

### Preoperative Examinations

For all patients, a chest radiograph, barium meal, Doppler ultrasound examination of the supraclavicular lymph nodes, and contrast enhanced computed tomography scan of the chest and abdomen were routinely administrated to evaluate the clinical stage of the tumor. Endoscopic ultrasonography (EUS) was also performed for most of these patients after the year 2010. Positron emission tomography (PET) was not routinely performed before surgery.

### Data Collection

All clinicopathological data and laboratory data were obtained from the patients’ medical records. Tumors were staged according to the 8th edition American Joint Committee on Cancer TNM staging system for ESCC. Weight and height were collected within 1 week before surgery. Alcohol drinking was divided into ever and never. An ever drinker was defined as a person who drank ≥1 time per week. BMI was calculated as follows: (weight, kg)/(height^2^, m^2^).

### Surgery

Most of the patients underwent esophagectomy through a right thoracotomy, while other patients underwent a left thoracotomy. For lymphadenectomy, the regional lymph nodes in the middle mediastinal, lower mediastinal, and upper abdominal regions were routinely dissected for all patients. For patients who underwent esophagectomy through a right thoracotomy, the lymph nodes around the left and right recurrent laryngeal nerves were also dissected.

### Statistical Analysis

Categorical variables were compared by the χ^2^ test or Fisher’s exact test. Overall survival (OS) and cancer-specific survival (CSS) were estimated using the Kaplan-Meier method, and the survival differences were assessed by the log-rank test. Multivariate Cox regression analysis was applied to identify independent prognostic factors, and all clinicopathological factors were included in the analysis due to the highly stable results. A receiver operating characteristic (ROC) curve was constructed to evaluate the sensitivity and specificity for 5-year OS, and the highest Youden’s index was used to identify the appropriate cutoff point for BMI. *P*< 0.05 was set to indicate significance. All statistical analyses were conducted in SPSS 20.0 software (IBM, Armonk, New York, USA).

## Results

### Patient Characteristics

Of the 817 patients with esophageal carcinoma who underwent esophagectomy between September 2014 and December 2017, 761 patients were diagnosed with ESCC. We excluded 116 patients who received neoadjuvant therapy (including 94 patients who received neoadjuvant chemoradiotherapy, 13 patients who received neoadjuvant radiotherapy, and 9 patients who received neoadjuvant chemotherapy) and 25 patients lacking any follow-up data, leaving 620 patients for analysis in this study. The study cohort included 229 ever drinkers and 391 never drinkers. Most patients were males (76.9%), and the median age was 61 years (range, 38 to 84 years). The mean number of lymph nodes dissected was 26.8 ± 11.0, with a median number of 26 (range, 6–74). Based on the 8th edition TNM staging system, 283 patients (45.6%) had pN0 disease, 207 patients (33.4%) had pN1 disease, 102 patients (16.5%) had pN2 disease, and 28 patients (4.5%) had pN3 disease. Radical resection was performed in 594 patients (95.8%), while palliative resection was performed in 26 patients (4.2%). The hospital mortality rate was 0.5% (3/620).

Twenty-two patients in this study had multiple primary malignancies (including 5 patients with synchronous malignancy and 17 patients with metachronous malignancy). The most common sites for multiple primary malignancies were head and neck in 10 cases, the esophagogastric junction in 5 cases, the lung in 3 cases, the stomach in 2 cases, the breast in 1 case, and the colon in 1 case.

### Cutoff Point for BMI

The median preoperative BMI was 20.90 kg/m^2^ (range, 13.30–32.70). One hundred seventeen patients were underweight (BMI <18.5 kg/m^2^), 448 patients were normal weight (BMI ≥ 18.5 to 24.9 kg/m^2^), and the other 55 patients were overweight or obese (BMI ≥ 25.0 kg/m^2^). To maximize the predictive value for BMI, we used the ROC curve to determine the appropriate cutoff point, which was 18.75 kg/m^2^ in this study. We further subdivided all the patients into two groups for analysis: the low BMI group (≤18.75 kg/m^2^) and the high BMI group (>18.75 kg/m^2^).

### Correlation Between Alcohol Consumption Status and Clinicopathological Factors

**Table 1** shows the correlation between alcohol consumption status and patient clinicopathological factors. Of the 229 ever drinkers, there was only one female patient (0.4%), which was a significantly lower percentage than that of female never drinkers (36.3%) (P<0.001). Ever drinkers were also found to have longer tumor lengths, advanced pT category disease and advanced pN category disease (P<0.05). Moreover, the tumors of ever drinkers were more often located in the lower third of the esophagus (P=0.012). However, there was no significant difference in BMI between ever drinkers and never drinkers.

**Table 1:** Correlation of alcohol drinking status with the clinicopathological features.

	No. Patients	Alcohol drinking		X^2^	*P* value
		Ever	Never		
Sex				104.7	<0.001
Male	477	228 (99.6%)	249 (63.7%)		
Female	143	1 (0.4%)	142 (36.3%)		
Age (yr)				0.033	0.857
≤60	303	113 (49.3%)	190 (48.6%)		
>60	317	116 (50.7%)	201 (51.4%)		
Tumor location				8.812	0.012
Upper third	110	40 (17.5%)	70 (17.9%)		
Middle third	388	130 (56.8%)	258 (66.0%)		
Lower third	122	59 (25.8%)	63 (16.1%)		
Tumor length				6.743	0.009
≤5cm	434	146 (63.8%)	288 (73.7%)		
>5cm	186	83 (36.2%)	103 (26.3%)		
Histologic grade				0.016	0.992
Well	210	78 (34.1%)	132 (33.8%)		
Moderate	322	119 (52.0%)	203 (51.9%)		
Poor	88	32 (14.0%)	56 (14.3%)		
BMI (kg/m^2^)				0.272	0.602
≤18.75	137	48 (21.0%)	89 (22.8%)		
>18.75	483	181 (79.0%)	302 (77.2%)		
Thoracotomy				1.103	0.294
Left	161	65 (28.4%)	96 (24.6%)		
Right	459	164 (71.6%)	295 (75.4%)		
Resection margin				0.990	0.320
Radical	594	217 (94.8%)	377 (96.4%)		
Palliative	26	12 (5.2%)	14 (3.6%)		
pT category				7.323	0.007
pT1-2	175	50 (21.8%)	125 (32.0%)		
pT3-4	445	179 (78.2%)	266 (68.0%)		
pN category				5.890	0.015
pN0	283	90 (39.3%)	193 (49.4%)		
pN1-3	337	139 (60.7%)	198 (50.6%)		

BMI, body mass index.

### Survival Analysis for the Entire Patient Cohort

The last follow-up was conducted in December 2020, with a mean follow-up time of 34.7 months (range, 1–69 months). Two hundred and fifteen patients died, and 10 patients were lost to follow-up (1.6%).

The 1-, 3- and 5-year OS rates for the entire group were 88.5%, 66.0% and 61.3%, respectively, and the 1-, 3- and 5-year CSS rates were 88.7%, 66.3% and 62.2%. In univariate analysis for OS, tumor length, BMI, thoracotomy, resection margin, pT category, and pN category were significantly correlated with survival (**Table 2**). Patients with high BMI had a significantly better OS than those with low BMI (63.5% vs. 53.5%, P=0.045). Although the 5-year OS of 57.1% for ever drinkers was lower than that of 63.8% for never drinkers, the difference was not significant (P=0.077). In univariate analysis for CSS, only tumor length, thoracotomy, resection margin, pT category, and pN category were significantly correlated with survival. Although the 5-year CSS of 55.4% for patients with low BMI was lower than that of 64.1% for patients with high BMI, the difference was not significant (P=0.069).

**Table 2:** Univariate analysis in regard to overall survival and cancer-specific survival according to clinicopathological factors.

Variable	5-yr OS (%)	*P* value	5-yr CSS(%)	*P* value
Sex		0.129		0.125
Male	59.4		60.2	
Female	67.6		68.8	
Age (yr)		0.946		0.709
≤60	61.1		61.1	
>60	61.6		63.4	
Tumor location		0.439		0.514
Upper third	60.5		60.5	
Middle third	62.5		63.4	
Lower third	56.7		58.0	
Tumor length		<0.001		<0.001
≤5cm	66.2		66.5	
>5cm	50.0		52.2	
Histologic grade		0.289		0.242
Well	64.4		66.0	
Moderate	59.0		59.7	
Poor	63.4		63.4	
Alcohol drinking		0.077		0.088
Ever	57.1		58.1	
Never	63.8		64.6	
BMI (kg/m^2^)		0.045		0.069
≤18.75	53.3		55.4	
>18.75	63.5		64.1	
Thoracotomy		0.005		0.005
Left thoracotomy	53.7		54.7	
Right thoracotomy	64.0		64.8	
Resection margin		<0.001		<0.001
Radical	62.9		63.8	
Palliative	21.8		21.8	
pT category		<0.001		<0.001
pT1-2	73.7		74.4	
pT3-4	56.3		57.3	
pN category		<0.001		<0.001
pN0	75.8		77.7	
pN1-3	49.3		49.3	

BMI, body mass index; CSS, cancer-specific survival; OS, overall survival.

In multivariate analysis for the entire group, only thoracotomy, resection margin, and pN category were found to be independent predictors for OS and CSS (**Table 3**). BMI and alcohol consumption were not independent prognostic factors for the entire group.

**Table 3:** Multivariate analysis in regard to overall survival and cancer-specific survival of the 620 patients with esophageal squamous cell carcinoma.

Prognostic factor	Hazard Ratio	95%CI	*P* value
Overall survival			
Sex	1.048	0.718-1.529	0.807
Age	0.908	0.691-1.192	0.486
Tumor location	1.083	0.850-1.379	0.520
Tumor length	1.295	0.963-1.741	0.087
Histologic grade	1.124	0.914-1.381	0.268
Alcohol drinking	1.053	0.776-1.430	0.739
BMI	1.365	0.997-1.867	0.052
Thoracotomy	0.638	0.473-0.860	0.003
Resection margin	3.911	2.333-6.556	<0.001
pT category	1.166	0.809-1.680	0.411
pN category	2.707	1.967-3.724	<0.001
Cancer-specific survival			
Sex	1.040	0.710-1.523	0.842
Age	0.873	0.663-1.150	0.335
Tumor location	1.075	0.842-1.374	0.560
Tumor length	1.247	0.924-1.682	0.149
Histologic grade	1.132	0.920-1.394	0.241
Alcohol drinking	1.035	0.760-1.409	0.826
BMI	1.345	0.978-1.848	0.068
Thoracotomy	0.629	0.465-0.850	0.003
Resection margin	4.076	2.427-6.847	<0.001
pT category	1.157	0.800-1.674	0.438
pN category	2.929	2.112-3.724	<0.001

CI, confidence interval; BMI, body mass index.

### Survival Analysis Stratified by Alcohol Drinking Status

**Figure 1:**
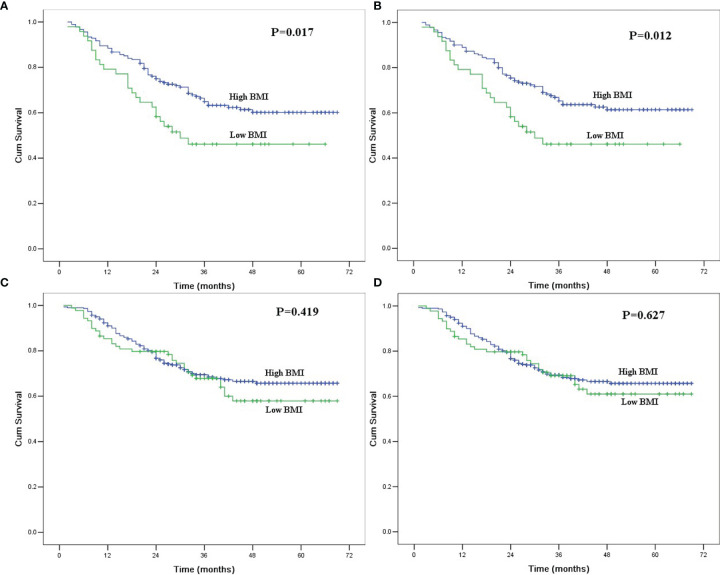
**(A)** Overall survival in ever drinkers with esophageal squamous cell carcinoma after esophagectomy according to body mass index. The difference was significant (P=0.017). **(B)** Cancer-specific survival in ever drinkers with esophageal squamous cell carcinoma after esophagectomy according to body mass index. The difference was significant (P=0.012). **(C)** Overall survival in never drinkers with esophageal squamous cell carcinoma after esophagectomy according to body mass index. The difference was not significant (P=0.419). **(D)** Cancer-specific survival in never drinkers with esophageal squamous cell carcinoma after esophagectomy according to body mass index. The difference was significant (P=0.627).

In univariate analysis for ever drinkers, age, tumor length, BMI, thoracotomy, resection margin, pT category, and pN category were found to be significantly correlated with OS and CSS (P<0.05). The 5-year OS and CSS for patients with low BMI were 46.1% and 46.1%, respectively, which were significantly lower than those for patients with high BMI (60.1% and 61.4%; P<0.05, **Figure 1**). In multivariate analysis for ever drinkers, low BMI was significantly correlated with worse OS (hazard ratio [HR] = 1.690; 95% confidence interval [CI], 1.037 to 2.756; P=0.035) and CSS (HR = 1.763; 95% CI, 1.077 to 2.886; P=0.024) than high BMI (**Table 4**).

**Table 4:** Overall survival and cancer-specific survival according to body mass index stratified by alcohol drinking status in multivariable models adjusted for other clinicopathological factors.

	Overall survival				Cancer-specific survival			
	5-yr OS (%)	*HR*	95% *CI*	*P* value	5-yr CSS (%)	*HR*	95% *CI*	*P* value
Never drinkers				0.251				0.391
Low-BMI	57.9	1.278	0.840-1.944		61.0	1.207	0.785-1.854	
High-BMI	65.7	Ref			65.7	Ref		
Ever drinkers				0.035				0.024
Low-BMI	46.1	1.690	1.037-2.756		46.1	1.763	1.077-2.886	
High-BMI	60.1	Ref			61.4	Ref		

BMI, body mass index; CI, confidence interval; CSS, cancer-specific survival; HR, hazard ratio; OS, overall survival.

In univariate analysis for never drinkers, tumor location, tumor length, thoracotomy, resection margin, pT category, and pN category were significantly correlated with OS and CSS (P<0.05). The 5-year OS and CSS for patients with low BMI were 57.9% and 61.0%, respectively, which were not significantly different from those for patients with high BMI (65.7% and 65.7%; P>0.05, **Figure 1**). In multivariate analysis for never drinkers, low BMI was not an independent predictor for worse OS (HR = 1.278; 95% CI, 0.840 to 1.944; P=0.251) or CSS (HR = 1.207; 95% CI, 0.785 to 1.854; P=0.391) compared with high BMI (**Table 4**).

## Discussion

Low BMI has been recognized as an epidemiological risk factor for ESCC (7). However, the prognostic value of BMI in ESCC patients is still controversial. Sun et al. (10) evaluated the data of 502 ESCC patients from Southern China and found that the 5-year OS rate of 25.2% for patients with BMI <18.5 was significantly lower than those of 46.1% and 48.1% for patients with BMI ≥ 18.5 to 24.9 kg/m^2^ and BMI ≥ 25.0 kg/m^2^ (P<0.001). Another study from Japan by Kamachi et al. (28) found that ESCC patients with BMI <18.5 kg/m^2^ had significantly worse OS and disease-free survival. However, Hasegawa et al. (12) found that OS and relapse-free survival were not significantly different in ESCC patients with different BMI levels. Duan et al. (14) found that high BMI worsened the long-term survival of ESCC patients after esophagectomy. The reasons for these inconsistent results may be attributed to the different cutoff values for BMI, the different number of patients and confounding factors, such as sex, age, smoking status (14, 29), or perhaps alcohol drinking status.

Alcohol consumption is one of the leading risk factors for cancer development and cancer death globally (30) and has been shown to affect BMI in the general population (19–21). A previous study found that alcohol abuse raised the risk of developing ESCC synergistically with low BMI (31). Therefore, it may be reasonable to assume that the prognostic value of BMI in patients with ESCC may be impacted by their alcohol drinking status. However, no previous studies have investigated the potential effect of the interaction between BMI and alcohol consumption on the prognosis of patients with ESCC. In our current study, we evaluated the prognostic value of BMI in a large cohort of ESCC patients who underwent surgical resection when stratified by alcohol drinking status. We found that BMI was a prognostic factor only for ever drinkers but not for never drinkers. After adjusting for other clinicopathological factors, patients with low BMI still had worse OS (HR = 1.690; 95% CI, 1.037 to 2.756; P=0.035) and CSS (HR = 1.763; 95% CI, 1.077 to 2.886; P=0.024) than patients with high BMI. However, in never drinkers, the 5-year OS and CSS were not significantly different between patients with low and high BMI in univariate and multivariate analyses. These results highlight the importance of alcohol drinking status in the prognostic prediction of BMI in ESCC patients and, for the first time, reveal that low BMI adversely impacts survival only in ever drinkers with ESCC but not in never drinkers.

The mechanism underlying the effect of the interaction between low BMI and alcohol consumption on the outcomes of ESCC patients is still not clear. We speculate that there are several possible explanations. First, malnutrition reflected in low BMI may be associated with a compromised immune system, which can lead to the development and early metastasis of cancer (32, 33). Immune cells in the tumor microenvironment may play an important role in regulating tumor progression (34). Malnourished patients have reduced lymphocyte function and cellular immunity, which leads to easier cancer development and metastasis (35). Chronic alcohol abuse also reduces cell-mediated immunity and lymphocyte numbers (36), which might enhance the adverse impact of malnutrition on the prognosis of cancer patients. Second, both ethanol and its metabolite acetaldehyde are carcinogenic to humans, which may lead to cancer development and metastasis (37). These effects may further be amplified by malnutrition, which is usually associated with insufficiency of micronutrients from improper maintenance of antioxidants and immune functions (31). Third, the presence of malnutrition might decrease the tolerance and response to treatment, which might also lead to the poor prognosis of cancer patients (38, 39). These effects may be amplified by alcohol consumption, as a previous study also showed that patients who consumed alcohol had poorer response rates to chemotherapy, smaller radiation doses, and less multimodality treatment (40). Based on these theories, whether a nutritional intervention and alcohol drinking cessation will improve the prognosis of these patients also needs to be investigated.

The strengths of our study are the relatively large patient cohort and homogeneity in histopathology and treatment. All of our patients chose esophagectomy as their initial treatment and were histopathologically diagnosed with ESCC. We excluded patients who received neoadjuvant therapy to avoid potential treatment-related BMI changes. Nevertheless, our study also has several limitations. First, it was a retrospective study from a single center, which undermined its power. Second, most of the patients who consumed alcohol in this study were male patients, with only one patient who consumed alcohol being female. Thus, we are unable to elucidate the interaction between low BMI and alcohol consumption in female patients with ESCC. Third, we did not consider the synergistic effect between alcohol and tobacco consumption in this study, as a previous study found that the prognostic value of BMI may be impacted by smoking status in patients with ESCC, and alcohol consumption and smoking are highly correlated behaviors (29). Further studies with larger cohorts are needed to confirm our results and investigate the mechanism underlying the effect of the interaction between low BMI and alcohol consumption on the prognosis of patients with ESCC.

In conclusion, our study showed that low BMI was independently associated with worse survival only in ever drinkers with ESCC but not in never drinkers. Further studies should be conducted to evaluate our findings and elucidate the mechanism underlying the effect of the interaction between BMI and alcohol consumption on the prognosis of patients with ESCC.

## Data Availability Statement

The raw data supporting the conclusions of this article will be made available by the authors, without undue reservation.

## Ethics Statement

The studies involving human participants were reviewed and approved by ethics committee of Shantou University Medical College Cancer Hospital. The patients/participants provided their written informed consent to participate in this study.

## Author Contributions

Y-pC designed the research and wrote part of the paper. S-bC analyzed the data and wrote part of the paper. D-tL wrote part of the paper. All authors contributed to the article and approved the submitted version.

## Funding

The Medical Scientific Research Foundation of Guangdong Province of China (B2019070).

## Conflict of Interest

The authors declare that the research was conducted in the absence of any commercial or financial relationships that could be construed as a potential conflict of interest.

## Publisher’s Note

All claims expressed in this article are solely those of the authors and do not necessarily represent those of their affiliated organizations, or those of the publisher, the editors and the reviewers. Any product that may be evaluated in this article, or claim that may be made by its manufacturer, is not guaranteed or endorsed by the publisher.
